# One step further: improving paediatric TB diagnosis through user-centred research approaches

**DOI:** 10.5588/ijtldopen.24.0389

**Published:** 2024-09-01

**Authors:** F.W. Basile, E.M. Bijker, M. Sekadde, S.E. Dorman, J.J. Ellner, N. Engel, M. Ruhwald, R. Song

**Affiliations:** ^1^ Department of Paediatrics, Oxford Vaccine Group, University of Oxford, Oxford, UK;; ^2^ Department of Paediatrics, Maastricht University Medical Center, MosaKids Children’s Hospital, Maastricht, the Netherlands;; ^3^ National TB and Leprosy Program, Uganda Ministry of Health, Kampala, Uganda;; ^4^ Medical University of South Carolina, Charleston, SC, USA;; ^5^ Department of Medicine, Rutgers New Jersey Medical School, Newark, NJ, USA;; ^6^ Department of Health, Ethics & Society, Maastricht University, Maastricht, the Netherlands;; ^7^ FIND, Geneva, Switzerland.

**Keywords:** paediatric TB, tuberculosis, diagnosis, user-centred research, qualitative, R&D; feasibility, implementation

Dear Editor,

In September 2023, the second high-level meeting of the United Nations General Assembly on fighting TB resulted in renewed commitments to ensure 90% of those with presumed TB receive a quality-assured diagnosis with a WHO diagnostic of high-quality standards.^1^ The first pillar of the End TB Strategy has long been calling for integrated care, with the patient at the centre of service delivery.^2^ The research community has further emphasised the importance of mixed-methods and people-centred approaches to evaluate and improve TB services, including quality-assured diagnosis,^3,4^ but little consideration has been given to children. However, in 2022, one in every eight people with active TB was a child or adolescent,^5^ and children under five are at the greatest risk of disease and complications.^6^ Diagnosis remains one of the main roadblocks along the paediatric TB cascade of care due to factors such as non-specific symptoms, paucibacillary disease and difficulties in specimen collection. Therefore, paediatric TB is often treated empirically, leading to both overtreatment and missed cases. Although there is more attention to childhood TB in global policy agendas and diagnostic research and development (R&D), age-appropriate user-centred approaches are urgently required to ensure that the resulting policies, programmes and technologies meet the complex needs of paediatric users and settings.^7^ Research efforts have focused on developing minimally invasive, accurate diagnostics suitable for point-of-care use. The release of the 2022 WHO Children and Adolescent TB Consolidated Guidelines marked significant progress in endorsing rapid molecular tests on stool and treatment decision algorithms.^8^ However, recommendations are often based on research settings that are not representative of the primary healthcare levels where most children initially access care. Operational research to better understand the preferences, obstacles, and needs related to paediatric TB diagnosis is a priority to improve clinical standards.^7^ Reflections on the type and diversity of data generated and used are key in the afterglow of the release of the third Roadmap towards ending TB in children and adolescents, highlighting the urgency of accessing quality paediatric TB diagnosis and addressing data gaps.^1^ This will require quantitative and qualitative data from diverse research and programmatic settings collected at various times. Understanding how healthcare providers navigate the diagnostic process in real-world settings and triangulating these insights with caregivers, children, and program implementers’ perspectives can inform diagnostics development and their introduction to differentiated child and adolescent TB services.^9^

A user-centred framework is essential for identifying the enablers for the adoption and sustainable use of quality-assured diagnostics (Figure). Currently, TB diagnostic R&D focuses on adults before extending to children. This is mainly due to the diagnostic complexity of paediatric TB and reference standards, whereas adults provide a clearer framework for the rapid and cost-effective development of new tools. However, to meet the unique demands of childhood TB diagnosis, it is crucial to integrate paediatric TB perspectives early in the R&D process.^10^ Validation studies are an important opportunity to gather evidence on user experiences and preferences and assess product usability, acceptability and feasibility. It is important to note that if user feedback were to impact test design and diagnostic performance, accuracy trials would have to restart or include a bridging study, again highlighting the need for early integration of such assessments. To be easily translatable, the evidence base should be gathered not only in highly specialised trial sites, but also in primary healthcare centres representative of the average settings for TB care globally. Early identification of implementation challenges will help to inform multicomponent intervention packages to reduce policy-practice gaps. User perspectives must represent the intended setting of use, and users are naturally prone to change over time and in response to changing needs, epidemiology, and available diagnostic options. A dynamic assessment in programmatic settings can inform novel technology adaptations to ensure the uptake, sustainability and scalability of diagnostic interventions.

**Figure.**
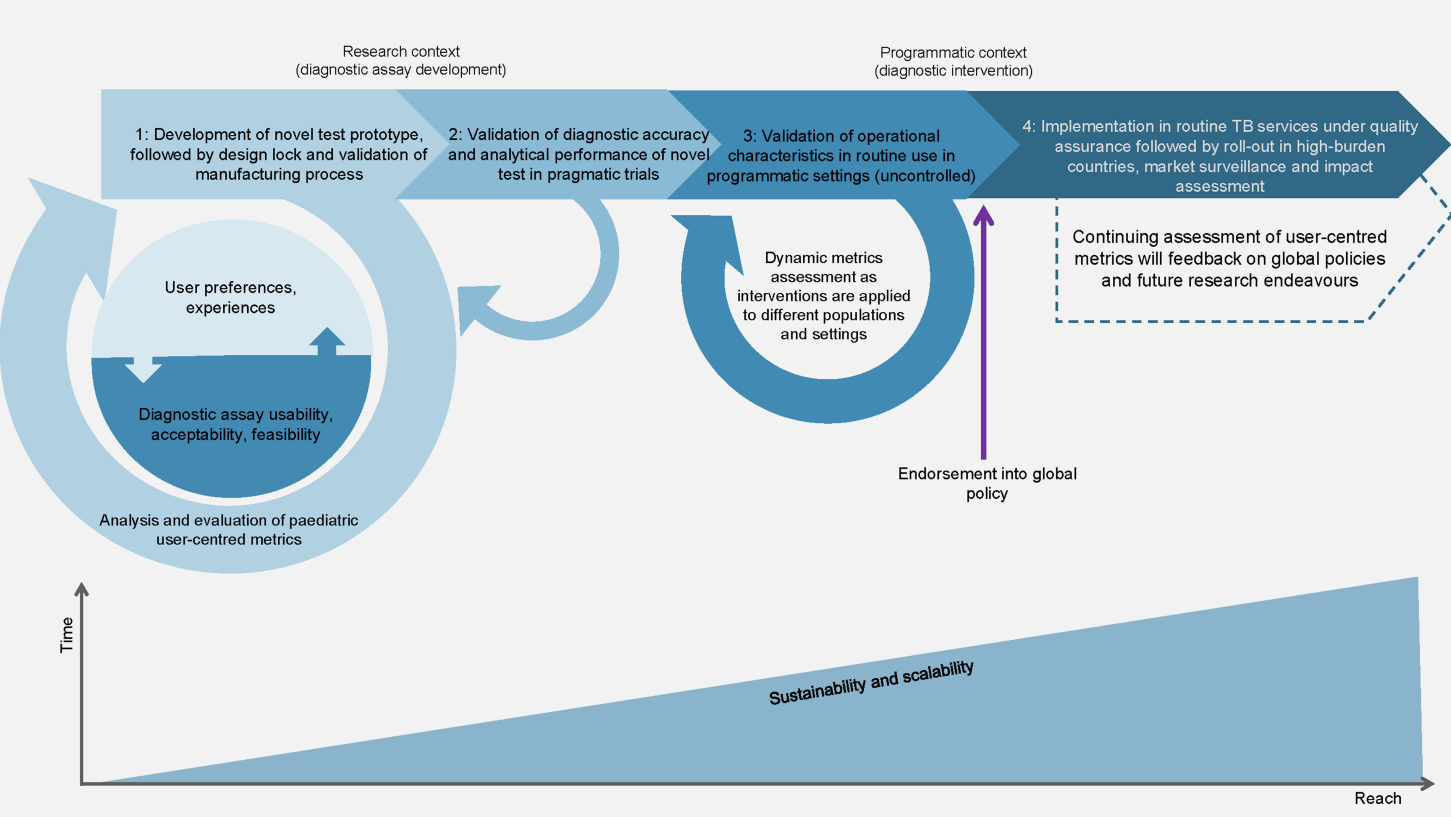
Conceptual framework for implementing novel diagnostic interventions in paediatric TB through user-centred research approaches. The framework emphasises the continuum between research and development and programmatic contexts and the interdependence of user-centred metrics. 1: The uptake of user-driven design in the early development stages (before design lock) is a key component of optimisation for improved usability, especially in paediatric TB, where invasive sampling and suboptimal accuracy hinder test uptake. User feedback should be collected throughout product validation; 2: To be easily translatable, the evidence base should be gathered not only in highly specialised trial sites but also in primary healthcare centres representative of the average settings for TB care globally; 3: Later operational research and economic evaluations will guide market entry, policy, and implementation of final products; 4: User perspectives require a dynamic assessment in relation with operational, geographical, financial, behavioural, and sociocultural aspects. In programmatic contexts, iterative feedback assessing stakeholders’ perspectives is critical to ensure that diagnostic interventions involving novel tests or approaches are both scalable and sustainable and to inform the delivery of appropriate intervention packages.

Ultimately, improved TB diagnosis in children will require the development of high-performance assays or child-friendlier sampling techniques while also ensuring that diagnostic aids are used in the best possible way where they are needed. The gap in qualitative research must be closed to achieve this ambitious but necessary goal. Shifting to a paradigm where evidence on user perspectives is routinely generated is particularly timely as the diagnostic pipeline for paediatric TB is richer than ever, and several consortia are researching novel, promising assays.^11^ Such a transition will require political commitment, research engagement and health system investments to foster environments for rapid adaptation and sustained quality improvements in paediatric TB care worldwide. TB researchers, technology developers, and policymakers must be receptive to those with lived experience, as well as healthcare providers and programme implementers. This will help create demand and resource mobilisation to ensure that the most appropriate, equitable, scalable and sustainable diagnostic interventions are available worldwide to children.
